# The association of obesity with eating disorders risk: online survey of a large cohort of Russian-speaking individuals seeking medical weight correction assistance

**DOI:** 10.1186/s40337-021-00456-y

**Published:** 2021-08-14

**Authors:** Grigory V. Rukavishnikov, Elena V. Verbitskaya, Olga Yu. Vekovischeva, Andrey V. Bobrovsky, Alexander O. Kibitov, Galina E. Mazo

**Affiliations:** 1 Department of Translational Psychiatry, V.M. Bekhterev National Medical Research Center for Psychiatry and Neurology, 3 Bekhterev Street, Saint-Petersburg, Russia 192019; 2 I.P. Pavlov First Saint-Petersburg State Medical University, 6-8 Lev Tolstoy St., Saint-Petersburg, Russia 197022; 3 Saint-Petersburg State University, 7-9 University Enb., Saint-Petersburg, Russia 199034; 4 V.P. Serbsky National Medical Research Centre on Psychiatry and Addictions, 23 Kropotkinskiy Lane, Moscow, Russia 119034

**Keywords:** Obesity, Abnormal eating behaviors, EAT-26, BMI, Online survey

## Abstract

**Background:**

Eating Disorders pose a serious health risk to individuals. Often, eating disorder symptoms are overlooked when assessing obesity risk. The current cross-sectional study was focused on the search of association between disordered eating behaviors evaluated by Eating Attitudes Test 26 (EAT-26) and obesity in a large cohort of Russian-speaking adults seeking online assistance with medical weight correction.

**Methods:**

The web-based cross-sectional study evaluated the data of online Eating Attitudes Test 26 (EAT-26) completed by 13,341 registered adult visitors of weight loss clinic website. The EAT-26 provides an overall score for potential eating disorders risk, as well as scores for three subscales: Bulimia, dieting, and oral control. Additional self-reported information about sex, weight, height, and age of respondents was used for analysis. The nonparametric analysis of variance and binominal logistic regression modeling were applied to search for an association between obesity and EAT-26 total score and subscales scores. The critical level of the significance was considered as α = 0.05.

**Results:**

Women (94%) had lower BMI values but higher EAT-26 total score than men, which was indicated as statistically significant by a Wilcoxon Signed-Ranks Test (*Z* = − 11.80, *p* < 0.0001). Logistic regression for the whole cohort revealed that Bulimia subscale score was associated with higher risk of obesity (OR = 1.03, 95% CI 1.02–1.05) whereas higher score of EAT-26 oral control subscale was associated with decreased risk of obesity (OR = 0.93, 95% CI 0.91–0.95). Separate analysis for men and women showed that in men higher obesity risk was associated with higher oral control subscale scores (OR = 1.08, 95% CI 1.06–1.11); while in women both dieting and bulimia subscales scores were associated with higher obesity risk (OR = 1.02, 95% CI 1.01–1.03 and OR = 1.03, 95% CI 1.02–1.05, respectively). Older age was associated with obesity risk for both women and men.

**Conclusions:**

In a large cohort of individuals seeking medical weight correction assistance, the risk of obesity was associated with the higher EAT-26 scores, age, and sex. Moreover, different eating disorder risk profiles were associated with obesity in men and women. Higher oral control subscale score was associated with decreased risk of obesity in women, but with higher risk in men. Older age was a shared obesity risk factor for both sexes. Therefore, the use of EAT-26 would facilitate individual diagnostic assessment for specific eating disorders in different sub-cohorts. Further assessment of separate EAT-26 subscales may be important to predict sex-/age-specific risks of obesity that implies their study in the future.

**Plain English summary:**

Obesity is a significant health problem. Different factors (e.g. social, biological, and behavioral) are important for their successful treatment. Abnormal eating behaviors may be one of the most likely predictors of increased body weight. This study aims to determine whether there is a significant association between obesity and scores on the eating behavior questionnaire-Eating Attitudes Test-26 (EAT-26)-in a large cohort of adults seeking medical weight correction assistance at a private weight loss clinic web-site. According to the study results, the association was shown for the male sex, older age, and higher Bulimia scores as measured on the EAT-26. Moreover, different EAT-26 scales were associated with obesity risks in women and men subgroups, while older age was a shared risk factor for obesity in both sexes. The findings may suggest sex-/age-specific diagnostic approach and treatment strategies for individuals with obesity.

## Background

Overweight and obesity are among the most significant health problem in the world that increase risks of various chronic diseases such as cardiovascular pathology, metabolic disorders, gastrointestinal dysfunction, and musculoskeletal pathology [1, 2]. According to recent epidemiological data, up to 39% (about 1.9 billion) of the world's adult population is overweight, and 13% (about 650 million) are suffering from obesity [1]. Obesity is a complex disease of multifaceted origin, including physiological, socioeconomic, biological, and behavioral factors [3–5]. Moreover, different types of disordered eating behavior patterns (binge eating, emotional eating, external eating, responding to food craving) are often associated with increased weight [6–8].

The self-reported Eating Attitude Test 26 (EAT-26) was chosen as one of the most reliable, common, and simple questionnaires to detect eating disorders risks in the human population [9–12]. The EAT-26 questionnaire has been used in Russia and in countries of the Commonwealth of Independent States (CIS) since the early 2000s [9, 13]. According to the recommendations of the Belarus Ministry of Health, the EAT-26 can be used with the intention to screen for eating disorders in different cohorts [9, 13]. However, numerous studies searching the association between eating disorders and obesity are focused on cohorts of limited age, such as adolescents or students, and the results remain understudied and controversial [14–19].

The individuals seeking medical weight correction assistance could be a fairly representative cohort to study the relationship between obesity and EAT-26 scores, as the treatment-seeking individuals are likely to be more aware of increased BMI and can report specific eating behaviors patterns. At the same time perception of self-weight in such individuals could be very variable and occasionally reflect conditions without the need for treatment [20]. Thus, it also seems relevant to assess the impact of sex and age on the association of weight and disordered eating behavior patterns in such a cohort. Moreover, previous findings showed that in treatment-seeking individuals with exceed BMI weight gain and loss were often associated with discorded eating behaviors, especially binge eating [21]. Because of that despite receiving proper treatment such patients could rapidly regain weight to even greater scores than initial ones, contributing to increased morbidity and mortality [21].

Therefore, further research is needed to identify the association between obesity and the occurrence of disordered eating behaviors, as well as to check whether they occur in individuals seeking medical weight correction assistance.

Our study aimed to determine whether there is a significant association between obesity and possible disordered eating behaviors measured with Eating Attitudes Test-26 in a large sample of individuals seeking medical weight correction assistance.

## Methods

### Participants

The online Eating Attitudes Test-26 (EAT-26) survey data has been collected between 2013 and 2018 from the adult Russian-speaking, medical weight correction seeking visitors of a private weight loss clinic website (https://doctorbormental.ru/).

The inclusion criteria were physical age (from 18 to 65 years), Russian Federation or CIS country citizenship, the ability to read, write and understand Russian, and signed consent to provide personal data for research purposes. Respondents were excluded from the analysis if they were outside the specified age range; had incomplete data (age, weight, height, CIS); or rejected the visitor’s agreement. Additional self-reported information about sex, weight, height, and age of respondents was used for analysis. Survey completion took no more than 20 min.

Data collection was secured on the clinic site. The data records contained 45,439 entries. However, 32,098 entries that did not meet the inclusion criteria (17,164 hid age/sex/weight/height info; 14,350 participants skipped items of the EAT; 120 put incorrect data (e.g. age = 8, and weight = 800 kg); 286 had age at < 18 or > 65), as well as duplicate entries (n = 3) were found), were excluded from the study. Also 78 participants (0.3% of the cohort) with BMI values below normal (BMI < 18.5) were excluded from statistical modeling due to their dissonance to the pre-specified study focus. Thus, the information from 13,341 respondents who lived in 8 administrative regions of the Russian Federation or at 5 CIS countries was analyzed.

The study was approved by the Institutional Review Board (IRB). Research principles of the human study were consistent with the general ethical principles of the World Medical Association Declaration of Helsinki (2013).

### Online survey

The online version of the Eating Attitude Test 26 (EAT-26) was used to detect risks of disordered eating behaviors. A cutoff score of 20 (inclusive) is considered to indicate the risk of an eating disorder and, therefore, the reason to recommend contact with a mental health specialist [10].

The Kettle’s method (kg/m^2^) was used to calculate BMI automatically [1]. The EAT-26 was divided categorically on three subscales: dieting (score range 0–39), Bulimia (score range 0–18) and oral control (score range 0–18). Total EAT-26 scores ≥ 20, suggested a risk of eating disorders [10].

**Fig. 1:**
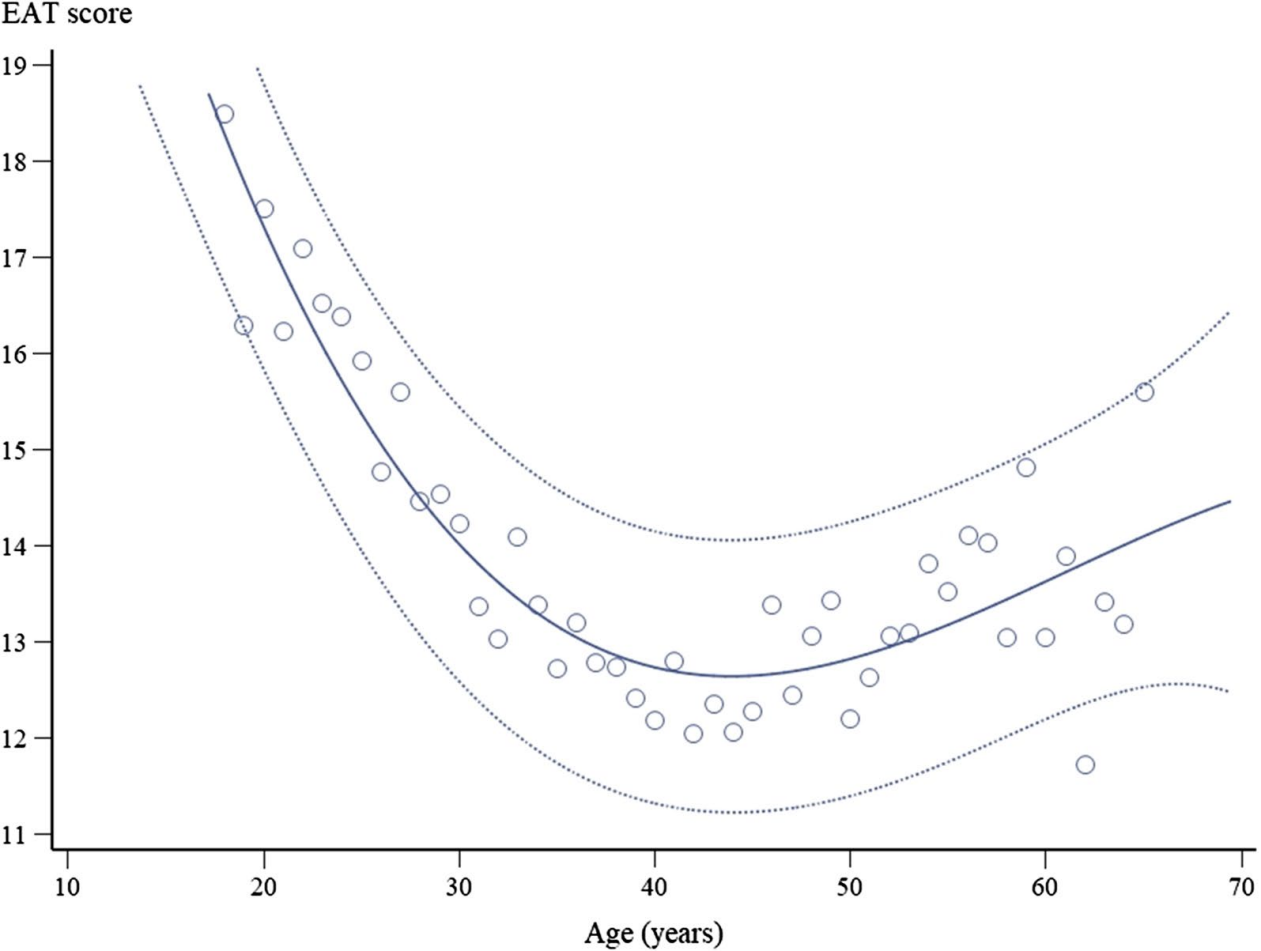
Scatter-plot of correlation between EAT-26 score and age

Preliminary statistical analysis revealed that EAT-26 scores and age had a non-linear relationship (Fig. 1), which led to the decision to convert age from a continuous measure into groups, for the purpose of statistical modeling. The categories chosen for age were: (1) 18–24 years old, (2) 25–44 years old, (3) 45–59 years old, and (4) above 60 years old in accordance with the international age classification criteria [22].

Similarly, for the analysis purposes, BMI was also converted from a continuous to a categorical variable, divided by WHO classification [1] into 2 categories: normal and Overweight with 18.5 ≤ BMI < 30 versus Obesity with BMI ≥ 30 (including Obesity I, Obesity II and Obesity III).

### Statistical analysis

Statistical analysis was performed using the statistical software package SAS 9.4. First, the distribution of independent variables was calculated as mean and standard deviation [Mean (SD)] or median and quartiles (Me) [Q1; Q3]. The critical level of the significance (null hypothesis rejection) was considered α = 0.05. The nonparametric analysis of variance and binominal logistic regression modeling were applied to search for an association between BMI and EAT-26 subscales scores. Finally, BMI, EAT-26 total score, and EAT-26 subscale scores were used as quantitative scales or categorical variables depending on the statistical test. The categorical variables such as age (4 groups), sex (2 groups), BMI (2 groups), and EAT-26 (2 groups) were presented as frequency and percentage (%) and were analyzed by cross-tab method with Pearson Chi-Square test (χ^2^) or with Fisher exact test to observe differences. The EAT-26 score was analyzed using (1) Wilcoxon Two-Sample Test (also known as Wilcoxon Signed-Ranks Test) to compare sex differences and (2) nonparametric analysis of variance (ANOVA; Kruskal–Wallis test) to estimate the influence of factors such as age, sex, and BMI. Bonferroni multiple comparisons test was used if the null hypothesis was rejected by p < 0.05 for post hoc comparisons. The analyses were repeated for the EAT-26 subscales Dieting, Bulimia, and Oral control. Several binomial logistic regressions were constructed to predict obesity (BMI ≥ 30) (yes/no) in the cohort by sex, age and EAT-26 subscale scores and their interactions. One regression model was built for the whole cohort and two separate regressions modelled for the female and the male subgroups separately.

## Results

### Age, sex and BMI characteristics of the cohort

Cohort composition for men and women by age, BMI and EAT-26 scores is presented in Table 1. The women differed from men in all measurements except for the oral control subscale of the EAT-26 (see column Wilcoxon Two-Sample Test).

**Table 1:** Cohort composition for men and women by age, BMI and EAT-26 scores

	Gender										All (N = 13,341, 100%)					Wilcoxon two-sample test
	Women (N = 12,628, 94.7%)					Men (N = 713, 5.3%)										
	Mean	SD	Median	Q1	Q3	Mean	SD	Median	Q1	Q3	Mean	SD	Median	Q1	Q3	
Age (years)	37.7	9.96	36	30	44	39.2	10.06	38	32	46	37.7	9.97	37	30	44	Z = 4.13; p < 0.0001
BMI	31.9	6.89	31.0	26.8	35.8	36.0	7.59	34.9	30.8	39.9	32.1	6.99	31.2	27	36	Z = 14.87; p < 0.0001
EAT score	13.8	8.57	12	7	19	10.3	8.32	9	4	14	13.58	8.6	12	7	19	Z = − 11.80; p < 0.0001
Dieting	10.3	6.06	10	6	14	7.9	5.85	7	3	12	10.2	6.08	10	5	14	Z = − 10.99; p < 0.0001
Bulimia	2.1	2.75	1	0	3	1.2	2.14	0	0	2	2.0	2.73	1	0	3	Z = − 9.98; p < 0.0001
Oral control	1.3	1.88	1	0	2	1.3	2.07	0	0	2	1.3	1.89	1	0	2	Z = − 1.07; p = 0.2848

^a^N—absolute number of the subjects, %—percentage of total cohort, SD—standard deviation, Q1 and Q3—the first(25%) and the third quartile

Cohort comparisons of BMI categories obesity and no obesity by age, BMI and EAT-26 scores (Table 2) indicated the BMI categories were similar by EAT-26 total score and EAT-26 dieting subscale score (see column Wilcoxon Two-Sample Test).

**Table 2:** Cohort composition for obesity and no obesity categories by age, BMI and EAT-26 scores

	BMI												Wilcoxon two-sample test
	Obesity (BMI ≥ 30)						No obesity (BMI < 30)						
	N	Mean	SD	Median	Q1	Q3	N	Mean	SD	Median	Q1	Q3	
Age	7687	39.64	10.16	38	32	47	5654	35.17	9.1	34	29	41	Z = − 24.84; p < 0.0001
EAT score	7687	13.42	8.26	12	7	18	5654	13.79	9.03	12	7	19	Z = 0.64; p = 0.521
Dieting	7687	10.06	5.92	10	6	14	5654	10.3	6.28	10	5	14	Z = 1.35; p = 0.1774
Bulimia	7687	2.06	2.67	1	0	3	5654	2	2.81	1	0	3	Z = − 3.84; p = 0.0001
Oral control	7687	1.19	1.82	0	0	2	5654	1.41	1.97	1	0	2	Z = 7.80; p < 0.0001

### Influences associated with BMI

#### Analysis of variance

Analysis of variance for BMI by age, sex, and EAT-26 score revealed that age and sex factors only were associated independently with BMI (F_3,13325_ = 29.99, p < 0.001 and F_1,13325_ = 40.89, p < 0.001, respectively). BMI was higher in men compared to women. Men of the youngest age category (18–24 years) had lower BMI than all other categories; 25–34 years men had lower BMI than 45–59 years and over 60 years men. 45–59 years and over 60 years categories of men showed similar BMI.

### Binomial logistic regression

Binominal logistic regressions were constructed to get how the age, sex, and EAT-26 score and their interactions may predict obesity (BMI >  = 30) outcome (yes/no).

The regression constructed for the whole cohort (Table 3) revealed that the higher EAT-26 Bulimia subscale score was associated with a higher risk of obesity (OR = 1.03, 95% CI 1.02–1.05) whereas a higher score of EAT-26 Oral control subscale was associated with decreased risk of obesity (OR = 0.93, 95% CI 0.91–0.95). The risk of obesity was higher in men (OR = 3.13, 95% CI 2.59–3.79) and in participants older than 24 years old: 25–44 years (OR = 1.95, 95% CI 1.7–2.23); 45–59 years (OR = 4.15, 95% CI 3.56–4.82); over 60 years (OR = 7.96, 95% CI 5.71–11.09). The classification power of the model was 62% (AUC = 0.623).

**Table 3:** Modeling effects of sex, age and EAT-26 scores on the BMI (obesity/no obesity) outcome (binominal logistic regression)

Parameter	Analysis of maximum likelihood estimates					Odds ratio estimates		
	DF	Estimate	Standard error	Wald chi-square	Pr > ChiSq	Point estimate	95% wald confidence limits	
							Min-	Max
Cohort								
Bulimia	1	0.034	0.007	24.729	< .0001	1.03	1.02	1.05
Oral control	1	− 0.073	0.010	56.210	< .0001	0.93	0.91	0.95
Men versus women	1	0.571	0.048	139.219	< .0001	3.13	2.59	3.79
Age 25–44 versus 18–24	1	− 0.374	0.046	64.806	< .0001	1.95	1.70	2.23
Age 45–59 versus 18–24	1	0.381	0.053	52.424	< .0001	4.15	3.56	4.82
Age > 60 versus 18–24	1	1.034	0.119	75.645	< .0001	7.96	5.71	11.09
Women								
Dieting	1	0.020	0.003	40.099	< .0001	1.02	1.01	1.03
Bulimia	1	0.030	0.008	15.461	< .0001	1.03	1.02	1.05
Oral control	1	− 0.069	0.011	42.945	< .0001	0.93	0.92	0.95
Age 25–44 versus 18–24	1	− 0.064	0.030	4.435	0.0352	2.46	2.16	2.81
Age 45–59 versus 18–24	1	0.617	0.043	209.672	< .0001	4.87	4.18	5.67
Age > 60 versus 18–24	1	0.412	0.083	24.421	< .0001	3.96	3.04	5.16
Men								
Oral control	1	0.080	0.013	37.186	< .0001	1.08	1.06	1.11
Age 25–44 versus 18–24	1	0.511	0.127	16.106	< .0001	5.75	3.10	10.68
Age 45–59 versus 18–24	1	1.198	0.205	34.150	< .0001	11.44	5.38	24.34
Age > 60 versus 18–24	1	− 0.469	0.302	2.422	0.1196	2.16	0.78	5.98

The regression constructed for the women-only revealed that the higher EAT-26 Bulimia and dieting subscale scores were associated with a higher risk of obesity (OR = 1.03, 95% CI 1.02–1.05 and OR = 1.02, 95% CI 1.01–1.03, respectively) whereas a higher score of EAT-26 oral control subscale was associated with decreased risk of obesity (OR = 0.93, 95% CI 0.92–0.95). In addition, the risk of obesity was higher in women of 45–59 years and over 60 years age categories compared to the youngest age category (OR = 4.87, 95% CI 4.18–5.67 and OR = 3.96, 95% CI 3.04–5.16, respectively). The classification power of the model was 61% (AUC = 0.607). The regression constructed for the men only revealed that the higher EAT-26 oral control subscale score was, on the contrary, associated with higher risk of obesity (OR = 1.08, 95% CI 1.06–1.11). The risk of obesity increased in men of 25–44 years and 44–59 years age categories as compared with the men of the youngest age category (OR = 5.75, 95% CI 3.10–10.68 and OR = 11.44, 95% CI 5.38–24.44, respectively). The classification power of the model was 58% (AUC = 0.575).

## Discussion

The association between BMI and EAT-26 score was performed on the cohort of Russian-speaking individuals who voluntarily sought online medical assistance for weight correction. Around 58% of our respondents had obesity, while only 30% of the general Russian population in 2012–2014 was reportedly classified overweight or having obesity [23]. To the best of our knowledge, there were no previous studies of obesity prevalence in weight correction-seeking individuals in a Russian population. However, our study showed a lower-than-average BMI index compared to other studies of individuals with obesity seeking weight loss treatment [24].

The fact that BMI was higher in men than in women, which contradicts the findings for the general Russian population [23], may suggest that men begin to seek medical assistance with a weight problem when the problem becomes severe. Also, similar findings of sex influence were found for treatment-seeking individuals with exceeding BMI, where men had higher BMI’s and were older than women [24]. The high ratio of females to males in our cohort is common for other studies of treatment-seeking individuals with exceeding BMI [20, 21, 24, 25].

The higher EAT-26 total score in women may indicate that the eating disorders are more common for the women [12, 26]. The sex differences of higher BMI and higher EAT-26 total score in women were demonstrated in Jamaican adolescents [19]. In contrast, the research in adolescents of urban secondary schools in Sarawak, Malaysia, shows opposite findings [16]. In the current study, BMI was higher in men while EAT-26 total score was higher in women.

The dividing for sex approach revealed an association between BMI was dependent on the ages of both women and men: the youngest showed lower weight (see Table 3). Thus, we assume the association between BMI and eating risk may be specific to men and women. On the other hand, previous results of disordered eating behaviors research in treatment-seeking individuals with obesity showed variable and even inconsistent results, from significantly higher to very low prevalence of disordered eating patterns than the general population [20, 21, 24, 25]. Perhaps, the different methodological approaches to identify disordered eating behaviors (diagnostic interview vs. self-reported questioner) might be a reason for such contradictions.

Another interesting finding of our study was different association of separate EAT-26 subscales with obesity risk in men and women. Thus, higher oral control subscale score was associated with decreased risk of obesity in woman, but higher risk in men. One of the possible explanations for that could be increased weight gain after the period of restrictions and control in men [21]. Considering the significant association of different EAT sub-scales in males and females, subscales of EAT-26 may be important in themselves to predict sex-specific risks of weight gain that implies their study in the future.

### Limitations

The study has several limitations. First, the study utilized a cross-sectional design, which means we could not track any changes in the relationship between eating disorder risk and changes in weight over time. Second, the data were received from self-selected people seeking weight loss assistance online (with a proportion of them either underweight or normal weight) limiting the ability to extrapolate results to the general population. Moreover, the usage of the online questioner with free access can lead to some issues. Beyond self-reporting errors (such as with height and weight), the quality of the data received was lowered and, therefore, severely limited the number of respondents included in the analysis. Also, the age was collected continuously and then transformed into categories, resulting in the loss of power (although not substantial considering the large sample size). Finally, the number of women in our study was much higher than men, which could also affect our results' generalizability.

## Conclusions

The study of the association between obesity and eating disorder risk (EAT-26 scores) in the adult Russian-speaking individuals seeking medical assistance for weight correction showed that the older males who scored higher on the Bulimia EAT-26 subscale were at potentially higher risk of developing obesity. Moreover, different eating disorder risk profiles were associated with obesity in men and women. Higher oral control subscale score was associated with decreased risk of obesity in women, but with higher risk in men. Older age was a shared obesity risk factor for both sexes. Therefore, the use of EAT-26 would facilitate individual diagnostic assessment for specific eating disorders in different sub-cohorts. Further assessment of separate EAT-26 subscales may be important to predict sex-/age-specific risks of obesity that implies their study in the future.

## Data Availability

The datasets used and/or analyzed during the current study are available from the corresponding author on reasonable request.

## Abbreviations

BMI: Body mass index
CI: Confidence interval
CIS: Commonwealth of independent states
EAT-26: Eating attitudes test-26
OR: Odds ratio
WHO: World Health Organization
